# Anticipated health effects and proposed countermeasures following the immediate introduction of telework in response to the spread of COVID‐19: The findings of a rapid health impact assessment in Japan

**DOI:** 10.1002/1348-9585.12198

**Published:** 2021-02-02

**Authors:** Tomohisa Nagata, Daisuke Ito, Masako Nagata, Ayumi Fujimoto, Ryotaro Ito, Kiminori Odagami, Shigeyuki Kajiki, Masamichi Uehara, Ichiro Oyama, Seitaro Dohi, Yoshihisa Fujino, Koji Mori

**Affiliations:** ^1^ Department of Occupational Health Practice and Management Institute of Industrial Ecological Sciences University of Occupational and Environmental Health, Japan Kitakyushu Japan; ^2^ Medical Office Hitachi Health Care Center Hitachi Ltd. Hitachi Japan; ^3^ HOYA CORPORATION Tokyo Japan; ^4^ Advanced Occupational health Research and Consulting (AORC), Ltd Kitakyushu Japan; ^5^ Brother Industries, Ltd Nagoya Japan; ^6^ Corporate ESH Asahi Kasei Corporation Tokyo Japan; ^7^ Mitsui Chemicals, Inc. Tokyo Japan; ^8^ Department of Environmental Epidemiology Institute of Industrial Ecological Sciences University of Occupational and Environmental Health, Japan Kitakyushu Japan

**Keywords:** COVID‐19, health impact assessment, telework, working from home

## Abstract

**Objectives:**

The health effects of telework, which was introduced extensively in the immediate context of the COVID‐19 pandemic crisis in Japan, on teleworkers, their families, and non‐teleworkers, are unknown. Accordingly, we developed a rapid health impact assessment (HIA) to evaluate positive and negative health effects of telework on these groups and recommended easily implementable countermeasures.

**Methods:**

Immediately after an emergency was declared in Japan, we implemented a rapid, five‐step HIA. We screened and categorized health effects of telework for the three above‐mentioned groups, extracting their content, directionality, and likelihood. Following a scoping exercise to determine the HIA’s overall implementation, five experienced occupational health physicians appraised and prioritized the screened items and added new items. We outlined specific countermeasures and disseminated the results on our website. A short‐term evaluation was conducted by three external occupational health physicians and three nurses.

**Results:**

Following screening and appraisal, 59, 29, and 27 items were listed for teleworkers, non‐teleworkers, and family members of teleworkers, respectively, covering work, lifestyle, disease and medical care, and home and community. Targeted countermeasures focused on the work environment, business management, communications, and lifestyles for teleworkers; safety and medical guidelines, work prioritization, and regular communication for non‐teleworkers; and shared responsibilities within families and communication outside families for family members of teleworkers.

**Conclusion:**

The HIA’s validity and the countermeasures’ practical applicability were confirmed by the external evaluators. They can be easily applied and adapted across diverse industries to mitigate the wider negative effects of telework and enhance its positive effects.

## 1 INTRODUCTION

The new coronavirus (SARS‐CoV‐2) infection was first reported in Japan on January 16, 2020,^1^ and in February 2020, it was named COVID‐19 by the World Health Organization. To minimize the increasing impacts of COVID‐19 on economic activities, social life, and medical care, and to prevent the collapse of medical services, the Japanese government officially declared an emergency situation in seven prefectures on April 8, 2020.^2^ Subsequently, on April 16, 2020,^3^ this state of emergency was extended to all remaining prefectures. Accordingly, members of the public were requested to refrain from leaving their homes, and businesses were asked to restrict the use of their facilities.

Prior to the declaration of the emergency, some businesses had already initiated a switch to telework for some of their activities. Regardless of company size, many business employees began to telework after the emergency was declared. In Japan, telework has mainly been discussed as a method of reducing working hours and preventing overwork. However, its spread within most industries has been limited, with the exception of a few industries such as information technology. The reason is that a number of business operators have introduced telework without adequately attending to technical issues such as the information technology environment and security. In addition, Japan's labor contracts are based on working hours in principle, and many operators have not yet made comprehensive arrangements relating to their personnel and labor systems for implementing telework.

Although telework entails several advantages, its health effects, explored mainly within studies conducted in Western contexts, are significant. They include musculoskeletal disorders as well as psychological impacts relating to isolation and stress as well as long working hours.^4^, ^5^ The findings of a survey conducted in Japan revealed that whereas there are merits associated with telework, such as improved productivity and a better family life‐work balance; managing working hours and occupational health and safety pose challenges.^6^

The introduction of telework in response to the recent emergency declaration encompasses enormous numbers of industries and diverse occupations. There was almost no time for preparation before its introduction, which was not dependent on employees’ deliberate choices of a work style. There is concern that telework may induce health effects far in excess of the anticipated occupational health and safety issues. While the introduction of telework by business operators in response to the spread of COVID‐19 is necessary, many issues need to be simultaneously addressed. Several responses to address health issues have been initiated, including measures to prevent the spread of infectious diseases and handling employees working overseas. In the current situation, because many issues have to be addressed immediately, using limited available resources, a policy tailored to the actual existing situation is urgently required. However, given the rapid introduction of telework in the absence of adequate preparation and insufficient scientific evidence, health issues associated with telework also require prioritization, and countermeasures to be implemented in the very short term need to be formulated.

A health impact assessment (HIA)^7^, ^8^, ^9^, ^10^, ^11^, ^12^ comprises a series of procedures for developing measures to reduce the negative impacts of a newly proposed activity, policy, or business and for promoting health benefits in advance. Such assessments can be conducted in situations where a wide range of health impacts are anticipated, even in the absence of sufficient information, because of the nature of the process for evaluating new measures. They allow scope for the modification or addition of measures based on the results of the HIA. Therefore, we conducted a HIA focusing on the introduction of telework associated with COVID‐19 to assess its effectiveness for estimating and prioritizing health effects and deciding on countermeasures. For this study, we performed a rapid HIA of limited duration in the immediate and urgent context of the introduction of telework and developed recommendations for addressing its short‐ and medium‐term health effects.

## 2 METHODS

We conducted a rapid assessment of various kinds of health effects that could be induced through the sudden introduction of telework because of the spread of COVID‐19. Following the general procedure for performing an HIA, we assessed health effects using a five‐step process: screening, scoping, appraisal, reporting, and monitoring/evaluation.

### 2.1 Screening

Screening entails a preliminary assessment of health and its social determinants. On March 31, 2020, a screening process was discussed within a steering group comprising five researchers, whom each have more than a decade of experience in the field of occupational medicine. Health effects change over time, so we selected a period of a few months commencing immediately after the introduction of telework. Two points required consideration: (a) the specific group whose health is impacted and (b) expected health effects. We used a screening tool^11^, ^12^ to assess expected health effects divided into categories that included individual lifestyle, illness, social/regional effects, living environment, work performance, and service accessibility/quality based on a socio‐environmental model of health.^13^, ^14^ We extracted the content, direction (positive/negative), and the possibility of health effects (definite, probable, speculative, and unknown). Because negative health effects generally tend to increase during an HIA, researchers should actively emphasize positive health effects. Health outcomes may be positive or negative for individuals in the same situation. For example, some smokers will smoke more, whereas others will smoke less during telework. In this case, health effects in both directions were listed.

### 2.2 Scoping

Scoping entailed determining the overall implementation of the HIA using measures of health effects extracted during the screening stage. The steering group discussed how to proceed with subsequent phases of the HIA: the appraisal (the responsibilities of the person in charge, the schedule, and the method of appraisal), reporting (how to select the items to be reported and how to make recommendations), and monitoring/evaluation (how to confirm the validity of the evaluation of health effects and whether the recommendations are applicable in practice).

### 2.3 Appraisal

The expected health effects of the proposed measures and their supporting evidence were assessed during the appraisal phase. The HIA covered not only the health impacts of telework in general but also the special current circumstances wherein insufficient preparation time has led to a mix of teleworkers and non‐teleworkers. Because the associated health effects are advancing, the completion of reporting within a brief period was an urgent requirement. Therefore, we decided to prioritize the opinions of currently active occupational physicians rather than attempting to conduct an exhaustive search for existing evidence.

Five Senior Occupational Health Physicians certified by Japan Society for Occupational Health were interviewed during the period April 13‐17, 2020. These interviews were aimed at eliciting their responses to the following three questions concerning the health effects identified during the screening phase. (a) Are there any health effects that should be added to the current list? (b) Have workers actually consulted them regarding any of the listed health effects, or have they heard indirectly that they are anxious? (c) Could they prioritize each health effect according to three levels (high, moderate, and low) to facilitate companies in considering how to respond? The lists of health effects developed by the steering group were shared with five occupational health physicians and their responses were elicited on (a) additional health effects for inclusion, (b) whether the workers had consulted an occupational physician or nurse about their particular health problems, and (c) their prioritization of health effects. All five physicians had a supervisory role relating to the occupational health team in each company and constantly communicated with the human resource managers and employers. We requested the five occupational health physicians to answer the above questions with inputs from human resource managers and employers. Workers’ views were elicited via the second question. We believed that the application of this methodology would enable rapid completion of the HIA and incorporation of the views of key stakeholders, namely, employers, employees, and human resource managers.

### 2.4 Reporting

The reporting step entailed reducing health disadvantages and maximizing health benefits as well as making recommendations, such as modifying measures and introducing additional measures, based on the previous appraisal of health effects. One researcher, and two research collaborators who are both Associate Occupational Health Physicians certified by Japan Society for Occupational Health prepared a draft report for human resources, general affairs, or occupational health staff. We included not only negative health effects but positive ones in this draft based on the results of appraisal. In addition, we actively selected measures that could be quickly and easily implemented, even if their health effects have been assigned low priority.

Based on the draft, the steering group along with two research collaborators made the recommendations that described and explained the health effects and provided specific countermeasures and practical examples with cited references. The recommendations were widely disseminated through the website of the Department of Occupational Health Practice and Management on May 1, 2020.

### 2.5 Monitoring/evaluation

The monitoring/evaluation process focused on assessing whether or not the HIA was practically applicable in the decision‐making process relating to policy development and in short‐term and medium‐term assessments of evident health effects conducted after the policy's implementation. This field study to assess the health effects of telework through an HIA did not target a specific business operator; rather, it was intended to serve more widely as a reference for many business operators and was conducted in light of the need for a short‐term evaluation. We solicited the impressions and views of occupational health staff working on‐site regarding the effectiveness of the measures. The validity and practical applicability of the recommendations published on the website were confirmed by three occupational health physicians and three occupational health nurses who were not involved in the HIA and who are all currently active. All occupational health staffs were informed and agreed with this study.

## 3 RESULTS

A rapid HIA was conducted with the aim of expediting dissemination of the results of the evaluation to the public. Screening and scoping exercises were conducted from March 31 to April 12, 2020, and the appraisal was conducted from April 13 to April 17. The HIA results were compiled and documented from April 18 to April 30, and were released to the public on May 1, 2020.

### 3.1 Screening

There was a consensus on the importance of the health effects of telework on three screened groups. These groups were workers who became teleworkers (“teleworkers”), workers who were not deemed eligible to telework and continued to operate at the workplace (“non‐teleworkers”), and family members of workers who became teleworkers (“family members of teleworkers”). For each group, the health effects were organized into four categories: work, lifestyle, disease and medical care, and home and community.

### 3.2 Scoping

The following consensus emerged from a discussion held among the steering group on the specific methods of appraisal, reporting, and monitoring/evaluation. The first was the view that this HIA focused specifically on health effects concerning employees who have begun to telework as a response to the crisis and not on the general health effects of telework. Second, health effects should be assessed in a context entailing the risk of infection and anxiety about it. Third, because this is an unprecedented event, the voices of active professionals in the field rather than the findings of previous studies are needed. Fourth, the prompt compilation of recommendations is extremely urgent. For these reasons, we decided to conduct an interview with experienced occupational physicians as appraisal.

Moreover, we decided to create a report containing recommendations that emerged during the researchers’ discussions, and to disseminate these recommendations to the public widely. To evaluate the practical applicability of these recommendations, we sought the opinions and feedback of occupational physicians (who were all Senior Occupational Health Physicians certified by Japan Society for Occupational Health) and occupational health nurses (who were all members of the Japan Society for Occupational Health), who had experienced occupational health practice since more than 10 years ago but who had not been involved in the HIA development process.

### 3.3 Appraisal

In addition to the items included in the screening list (a total of 94 items), the final list contained 115 items. They were 59 items for teleworkers in Table 1, 29 items for non‐teleworkers in Table 2, and 27 items for family members of teleworkers in Table 3.

**TABLE 1:** The results of health impact assessment for teleworkers

List of health effect	Screening	Appraisal						Reporting
	Positive/negative	Number of voices^a^	Priority evaluated by five Senior Occupational Health Physicians					Items adopted as recommendations
			Physician 1	Physician 2	Physician 3	Physician 4	Physician 5	
Work								
Increased strain on eyes/shoulders/back caused by a suboptimal VDT environment	Negative	3	High	High	High	High	High	✓
Loss of concentration	Negative	1	Moderate	Moderate	Moderate	Moderate	Moderate	
The ability to work in a relaxed manner	Positive	0	Moderate	Low	Moderate	Moderate	Moderate	
Work must be intermittent	Positive	0	Moderate	Low	Moderate	Moderate	Moderate	
Work will be intermittent	Negative	2	Moderate	High	Moderate	Moderate	Moderate	✓
Less meeting time and less burden	Negative	0	Moderate	Low	Moderate	Moderate	Moderate	✓
More meeting time and increased burden	Negative	1	—	—	Moderate	—	Moderate	✓
Longer working hours (for meticulous and committed employees)	Negative	2	High	Moderate	High	Moderate	High	✓
Fatigue from not taking adequate breaks	Negative	1	—	—	—	High	Moderate	✓
Increased workload from providing support when a colleague is infected in the workplace	Negative	0	—	High	—	—	High	
Telework is a more efficient way of doing business	Positive	0	—	—	—	Moderate	Moderate	✓
Reduced support from supervisors and colleagues	Negative	2	Moderate	High	Moderate	Moderate	moderate	✓
Poor relationships with bosses and co—workers	Negative	0	—	—	—	Moderate	moderate	✓
Decreased labor productivity caused by difficulties in internal communication	Negative	3	High	High	High	High	high	✓
Decreased labor productivity caused by difficulties in communicating with people outside the company	Negative	1	High	High	Low	Moderate	high	✓
Feelings of isolation because of reduced communication opportunities in the workplace (for people living alone)	Negative	4	High	High	Moderate	High	high	✓
Work becomes more visible and progress is monitored	Positive	0	Moderate	Low	Moderate	Moderate	moderate	
Stress caused by changes associated with the introduction of a new system	Negative	2	High	High	Moderate	High	high	✓
The risk of information leakage when employees start teleworking in the absence of adequate security measures	Negative	0	—	—	High	—	high	✓
Deterioration in operational efficiency caused by a deteriorating communication environment (communication congestion)	Negative	1	—	High	—	—	high	✓
Decreased health and safety management (in the absence of supervision)	Negative	1	High	Moderate	Low	Moderate	moderate	
Lifestyle								
Decreased physical activity	Negative	5	High	High	High	Moderate	High	✓
Increased physical activity (from increased free time resulting from the elimination of commuting time)		1	—	—	High	—	Moderate	
Stiff shoulders and back pain caused by prolonged sitting	Negative	5	High	High	High	High	High	✓
Disruption of the rhythm of life (and especially the rhythm of sleep)	Negative	1	Moderate	Moderate	Moderate	Moderate	Moderate	✓
More sleep (from increased free time resulting from the elimination of commuting)	Positive	1	—	—	Moderate	—	High	
Elimination of commuting time/reduced stress on the commute	Positive	1	Moderate	Low	Moderate	Moderate	Moderate	✓
Reduction in traffic and work‐related accidents on the commute	Positive	0	Moderate	Low	Low	Low	Low	
Changes in the content of lunches, such as not using the company cafeteria	Negative	4	High	High	Low	Moderate	Moderate	✓
Poor diet	Negative	1	High	Unknown	Moderate	Moderate	Moderate	✓
Snacking at work	Negative	0	—	—	—	Low	Low	
Increase in the number of cigarettes smoked	Negative	0	Moderate	Moderate	Moderate	Moderate	Moderate	✓
Decrease in the number of cigarettes smoked	Positive	0	Moderate	Low	Moderate	Moderate	Moderate	✓
Increased alcohol consumption	Negative	0	Low	High	Moderate	Moderate	High	✓
Decreased alcohol consumption	Positive	0	Low	Low	Moderate	Low	Low	✓
Decreased entertainment (eg, drinking parties)	Negative	1	Low	Low	Low	Moderate	Low	
Disease and medical care								
Reduced risk of infection	Positive	1	High	Low	High	High	High	✓
Reduced fear of contracting an infectious disease	Positive	1	High	Low	Low	Moderate	Moderate	✓
Increased hygiene awareness and practices	Positive	0	Moderate	Low	Low	Low	Low	
Increased anxiety from being in a place without people	Negative	0	Moderate	Moderate	Moderate	Moderate	Moderate	
Overlooking of personal changes (worsening of depressive symptoms)	Negative	0	Moderate	High	High	High	High	✓
Progression of dementia	Negative	0	Low	Low	Low	Low	Low	
Increased stress	Negative	2	High	Unknown	High	Moderate	High	✓
Decreased motivation	Negative	1	Low	High	High	Moderate	High	✓
More personal time during the day and easier access to medical facilities	Positive	0	Moderate	Low	Moderate	Moderate	Low	
Medical collapse makes it harder to see a doctor	Negative	2	High	High	Moderate	Moderate	High	✓
Lower likelihood of being attended to at a medical facility near the workplace	Negative	1	—	—	Moderate	—	Moderate	✓
Home and community								
Increased opportunities for communication with family members (if living with one or more family members)	Positive	2	Moderate	Low	Low	Moderate	Moderate	✓
More time spent at home	Positive	0	Moderate	Low	Moderate	Moderate	Moderate	✓
Increased irritability from having less time alone (if living with family members)	Negative	0	Moderate	High	Low	Moderate	Moderate	
More divorces	Negative	0	—	—	Moderate	Moderate	Moderate	
Increase in domestic violence	Negative	0	High	Moderate	Moderate	Low	High	
Deterioration of the educational environment	Negative	1	High	Moderate	High	Moderate	High	
More people playing games	Negative	0	Low	Low	Moderate	Moderate	Moderate	
Not being able to go out to perform leisure activities	Negative	1	Moderate	Moderate	High	High	High	
Decrease in commuting costs	Positive	1	Moderate	Low	Low	Moderate	Low	
No more overtime pay and adverse impact on the family's finances	Negative	0	—	—	Moderate	Moderate	Moderate	
Reduced salaries	Negative	1	High	Moderate	High	Moderate	Moderate	
Reduced involvement with the local community	Negative	0	—	—	Moderate	Moderate	Moderate	

Note

VDT, video display terminal.

^a^ The number of the Senior Occupational Health Physicians certified by Japan Society for Occupational Health who were consulted by their employees worried about health effects.

**TABLE 2:** The results of health impact assessment for non‐teleworkers (workers who are not eligible for telework and continue to work at the workplace)

List of health effect	Screening	Appraisal						Reporting
	Positive/negative	Number of voices^a^	Priority evaluated by five Senior Occupational Health Physicians					Items adopted as recommendations
			Physician 1	Physician 2	Physician 3	Physician 4	Physician 5	
Work								
Frustration and anxiety about not being considered eligible for telework	Negative	3	High	High	High	High	High	✓
Change in concentration levels	Negative	0	Moderate	Moderate	Moderate	Moderate	Moderate	
The ability to work in a relaxed manner	Positive	0	Low	Low	Low	Moderate	High	
Less meeting time and less burden	Positive	0	Moderate	Low	Moderate	Moderate	Low	✓
Longer working hours	Negative	0	High	Moderate	Moderate	Moderate	Moderate	✓
Increased workload from providing support when a colleague is infected in the workplace	Negative	0	—	High	High	—	High	✓
Increased handling of non‐electronic data and miscellaneous duties, such as answering the telephone	Negative	0	—	—	—	—	—	
Decreased support from supervisors and colleagues	Negative	2	High	High	High	Moderate	High	✓
Decreased labor productivity because of poor communication	Negative	2	High	High	High	High	High	✓
Work becomes more visible and progress is monitored	Positive	0	Low	Low	Low	Low	Moderate	
Stress caused by changes associated with the introduction of a new system	Negative	1	High	High	Moderate	Moderate	Moderate	✓
Decreased health and safety management (in the absence of supervision)	Negative	0	Moderate	Moderate	Moderate	Moderate	Moderate	
Lifestyle								
Increase in the number of cigarettes smoked	Negative	0	Low	Moderate	Moderate	Moderate	Moderate	
Poor diet	Negative	0	Low	Unknown	Low	Moderate	Low	
Reduced wait time for lunch and more eating options	Positive	1	—	—	—	—	Moderate	
Disease and medical care								
Infection	Negative	1	High	High	Moderate	Moderate	High	✓
Fear of contracting an infection	Negative	4	High	High	High	High	High	✓
Increased hygiene awareness and practices	Positive	0	Moderate	Low	Moderate	Moderate	Moderate	
Increased anxiety from being in a place without people	Negative	1	High	Moderate	Moderate	Moderate	Moderate	
Overlooking of personal changes (worsening of depressive symptoms)	Negative	1	High	High	High	Moderate	High	✓
Increased stress	Negative	1	High	Unknown	High	Moderate	Moderate	
Decreased motivation	Negative	0	Moderate	High	Moderate	Moderate	Moderate	
Medical collapse makes it harder to see a doctor	Negative	1	High	High	High	Moderate	High	✓
Home and Community								
Decreased entertainment	Negative	1	High	Low	Moderate	Moderate	High	
Not being able to go out to perform leisure activities	Negative	1	High	Moderate	High	High	High	
Ability to come home early and talk more with family members	Positive	1	—	—	—	—	Moderate	
Decreased involvement in the community	Negative	0	—	—	Low	Low	Low	
Reduced salaries	Negative	0	High	Low	High	Moderate	Moderate	
Deterioration of the educational environment	Negative	0	High	Low	High	Low	High	

^a^ The number of the Senior Occupational Health Physicians certified by Japan Society for Occupational Health who were consulted by their employees worried about health effects.

**TABLE 3:** The results of health impact assessment for family members of teleworkers

List of health effect	Screening	Appraisal						Items adopted as recommendations
	Positive/negative	Number of voices^a^	Priority evaluated by five Senior Occupational Health Physicians					
			Physician 1	Physician 2	Physician 3	Physician 4	Physician 5	
Lifestyle								
Disruptions in the rhythm of life (and especially in the rhythm of sleep)	Negative	0	Moderate	Moderate	Moderate	Low	Moderate	
Passive smoking attributed to spousal smoking	Negative	0	High	Moderate	Moderate	Moderate	Moderate	✓
Increased drinking (more opportunities to drink with spouse)	Negative	1	Low	Low	Moderate	Moderate	Moderate	✓
Poor diet.	Negative	0	High	Unknown	Low	Moderate	Moderate	
Disease and medical care								
Reduced risk of infection	Positive	0	High	Low	Low	Low	Low	✓
Reduced fear of contracting an infectious disease	Positive	0	High	Low	High	Low	Moderate	✓
Increased hygiene awareness and practices	Positive	0	Moderate	Low	Low	Moderate	Moderate	
Increased anxiety from being in a place without people	Negative	0	High	Low	Low	Low	High	
Overlooking of personal changes (worsening of depressive symptoms)	Negative	0	Low	High	High	Low	High	
Progression of dementia	Negative	0	Low	Low	Low	Low	Low	
Increased stress	Negative	1	High	Unknown	Moderate	Moderate	High	
Decreased motivation	Negative	0	Low	High	Moderate	Moderate	Moderate	
Home and Community								
Increased household burden (from assuming spouse's share of household chores)	Negative	0	High	Low	Moderate	Moderate	Moderate	✓
Decreased burden of household chores (spouse shares in household chores)	Positive	0	Moderate	Low	Moderate	Moderate	Moderate	
Spend more time with spouse (a positive effect)	Positive	0	Moderate	Low	Low	Moderate	Moderate	
Spend more time with spouse (a negative effect)	Negative	1	High	Moderate	Low	Moderate	Moderate	
Increased opportunities to communicate with spouse	Positive	0	Low	Low	Low	Moderate	Moderate	
More time spent at home	Positive	0	Moderate	Low	Moderate	Moderate	Moderate	
Frustration from having less time alone	Negative	3	High	Moderate	High	Moderate	High	
Difficult to relax when family members are working	Negative	0	—	—	Moderate	Moderate	Moderate	
More people playing games	Negative	0	Low	Low	Moderate	Moderate	Moderate	
More divorces	Negative	0	—	—	Moderate	Moderate	Moderate	
Increase in domestic violence	Negative	0	High	Moderate	Moderate	Low	High	
Not being able to perform leisure activities	Negative	1	Moderate	High	High	High	High	✓
Decrease in commuting costs	Positive	0	Moderate	Low	Low	Low	Low	
Decline in community involvement	Negative	0	—	—	Moderate	Moderate	Moderate	✓
Deterioration of the educational environment	Negative	0	High	Moderate	High	Moderate	High	

^a^ The number of the Senior Occupational Health Physicians certified by Japan Society for Occupational Health who were consulted by their employees worried about health effects.

Whereas teleworkers experienced a reduced risk of COVID‐19 infection, several high‐priority health effects were identified. These included eye/shoulders/back strain, stress caused by changes associated with the introduction of a new system, isolation caused by reduced communication, overlooking of personal changes, and reduced work productivity. For non‐teleworkers, high‐priority items were fear of contracting COVID‐19 and frustration and anxiety about not being considered eligible for telework. A high‐priority item for family members of teleworkers was not being able to go out to perform leisure activities.

### 3.4 Reporting

Following discussions held among the steering group and two research collaborators, we formulated recommendations relating to health effects for teleworkers, non‐teleworkers, and family members of teleworkers (Table 4). All of the recommendations have been published in Japanese on the website of the Department of Occupational Health Practice and Management.^15^

**TABLE 4:** The proposed countermeasures for enhancing health and minimizing adverse health effects in the reporting of health impact assessments of teleworkers, non‐teleworkers, and family members of teleworkers

1. Teleworkers
Work
There are concerns about lost productivity resulting from poor communication.
Clarify individual job descriptions and the assignment of tasks.
It is important to have short, regular meetings at the beginning and end of the work day to identify and share work‐related concerns.
The chat functions of apps should be used to facilitate communication.
Changes in working hours and a lack of breaks are likely to occur.
Manage working hours objectively (eg, report start and end times and introduce tools to manage working hours).
Consider introducing a flextime or a deemed work hour system so that employees have the flexibility to change their scheduled work hours to accommodate their personal work, such as childcare or nursing care responsibilities.
A short time spent away from work is acceptable. It should be borne in mind that a normal work schedule also includes adequate rest.
All of the personnel in the workplace should be informed that they should refrain from asking colleagues to handle work during breaks or outside of office hours.
There are concerns about the stress induced by the introduction of a new system and the associated changes.
Create a simple manual on how to operate the system and distribute the instructions to the staff.
There are concerns that it will be difficult for supervisors to manage their subordinates.
Check in and talk to your employees more frequently than you previously did. Some companies have held more one‐on‐one meetings than usual to help employees stay motivated and to encourage them to take care of their physical and mental health.
Shoulder and back pain associated with telework is a concern.
Distribute checklists on appropriate features of office environments and educate employees on the correct working environment and when to take breaks.
Some companies provide desks and chairs for those wishing to take a break.
IT security and the risk of information leakage is a concern.
There is a concern that productivity may decline as a result of a deteriorating Internet environment and its effect on communication.
Monitor and support workers' Internet environments.
There are concerns about a reduction in work productivity (presenteeism) caused by health problems.
Changing the way we work is expected to improve the company's efficiency.
It is important to consider how to improve the efficiency of an entire department and not just that of an individual.
Best practices regarding business efficiency should be compiled within each department and shared with other departments.
There could be changes in meeting times.
Clarify the agenda and keep it as simple as possible.
Set rules for meeting times and durations based on the meeting size. Set a timer for presentations and discussions or have a supervisor take the lead in adjourning the meeting.
Lifestyle
A decrease in physical activity is a concern.
Educate people about the importance of exercising at home.
There is a lot of information on home‐based exercise available on the Internet.
Some companies have their staff perform exercises together listening to radio shows during video conferences held in the morning or evening.
Encourage workers to exercise in a way that avoids the “3Cs” (closed spaces, crowded places, and close‐contact settings). When exercising outside, people are encouraged to maintain as much distance from each other as possible. Studies recommend maintaining a distance of about 10 meters for jogging and about 5 meters for walking.
Lifestyle changes (eg, diet, drinking, and smoking) are anticipated.
Educate people to follow a well‐balanced diet rather than eating only prepared and instant foods to maintain their immunity against COVID‐19.
When drinking alcohol, set a time for drinking and the quantity of alcohol that will be consumed in advance and record them using an app or other means. This practice will make individuals aware of how much they are drinking and encourage them to reduce the amount that they drink.
WHO has suggested that smokers may be at a higher risk of contracting COVID‐19 and becoming seriously ill. Communicate strongly the need to reduce the number of cigarettes smoked and to quit smoking, and consider restricting the use of smoking rooms as a “3C” measure.
A change in life routines is to be expected.
Encourage workers to establish a structured routine by setting regular rising, bedtime, and meal times.
Scheduling online meetings in the mornings and evenings at fixed times and introducing a daily life record sheet are also effective practices.
Reducing commuting time and the stress of the commute are anticipated.
Disease and medical care
It is expected that the ease of access to medical care and disease control practices will change.
Educate people to continue to see their doctors on a regular basis so that they do not, on their own, discontinue hospital visits, and taking their medication. Online treatment may be available in some cases.
It is expected that the risk as well as the fear of infection will be reduced.
Home and community
A change in inter‐family relationships is to be expected.
2. Non‐teleworkers
Work
Frustration or anxiety about not being eligible for telework is a concern.
If you have specific complaints or concerns, please consult us about environmental improvements and consider implementing individual improvement measures.
There are concerns about lost productivity resulting from poor communication.
Implement infection control measures, but be proactive about communicating with each other.
There may be changes in the workload.
Prioritize your work.
There could be changes in meeting times.
Be clear about the agenda of meetings, and be punctual about wrapping them up. By carefully selecting participants, you can reduce the burden on them.
Stress from changes, such as the introduction of new IT systems, is a concern
Disease and medical care
Office workers have a higher risk of getting infected with COVID‐19 compared with teleworkers.
Workers who are at high risk of severe illness from COVID‐19 require attention.
The attention required by each worker will vary according to their health status. First of all, a policy for dealing with workers who are likely to become seriously ill should be established and disseminated.
There is increased concern about the high risk of contracting COVID‐19.
In addition to the fear of becoming infected, workers are also afraid that they may infect their families. It is important to listen to the workers expressing anxiety and to consider whether it is possible to accommodate some of their concerns.
Home and community
There is heightened fear among employees about infecting their families.
We recommend that you talk to them several times about their concerns. Please consider addressing some of their concerns at the workplace according to their situation and their own and their families’ wishes.
3. Family members of teleworkers
Lifestyle
There may be an increase in the burden of household chores.
Divide up household chores and try to work together within the family to get them done.
It is also useful to avail of housekeeping services, such as cleaning, laundry, and cooking.
Increased alcohol consumption is a concern.
Decide in advance what time you will drink alcohol and the amount of alcohol you will consume, and try to maintain appropriate drinking habits within your family.
An increase in passive smoking is a concern.
The most effective way of persuading people to quit smoking is to emphasize that doing so will benefit the health of their family members.
Home and community
Increased stressors result from the inability to go out.
Find stress‐relieving activities that your family can do at home, such as stretching, and watching movies.
Decreased community involvement is a concern.
It is also useful to create opportunities for people to get in touch with others and to connect with the wider society through phone calls, emails, and so forth.
Disease and medical care
The risk of COVID‐19 infection is reduced, with a concomitant reduction in anxiety about becoming infected.

#### 3.4.1 Teleworkers

Various recommendations targeted teleworkers, of which major parts were work related. We recommended to distribute a checklist for promoting self‐education on an appropriate work environment and how to take breaks, adding, as a reference.^16^ We also recommended improvement of the communication environment with security measures because of the risk of information leakage.

Our concern relating to time management was that the life‐work boundary would become unclear and teleworkers would not take sufficient breaks. As a countermeasure, we recommended that companies consider implementing objective task management, providing for short breaks, and encouraging sufficient rest.

A communication‐related concern was that supervisors would find it difficult to manage their subordinates’ work. We recommended that companies consider introducing teams to information‐sharing and objective work management tools and conduct regular physical and mental health checkups of their subordinates.

For health effects that were not related to work, there were concerns about a decrease in physical activity and changes in lifestyle (dietary habits, drinking, smoking, etc) that included both positive and negative effects as well as changes in lifestyle rhythm. To counter decreased physical activity, we will focus on educating people about the importance of exercising at home, pointing to the wealth of information on home‐based exercise available on the Internet (eg, the exercises recommended by the Sapporo City Sports Association).^17^

#### 3.4.2 Non‐teleworkers

It is conceivable that employees at the workplace are exposed to the risk of COVID‐19 infection at work or while commuting, and may be dissatisfied or anxious about working in that environment. Their anxiety is not only about being infected themselves but also about transmitting the infection to their families. Therefore, the voices of employees at a workplace must be heard, and sufficient measures must be taken to reduce the risk of infection.

In the area of disease and medical care, special care is required for individuals who are at particular risk of suffering severe symptoms if they contract COVID‐19 infections. They include older individuals; those with underlying diseases, such as diabetes, heart failure, and respiratory diseases; and individuals undergoing treatments like dialysis or taking immunosuppressive agents and anti‐cancer drugs. Therefore, we recommended the establishment of action‐based guidelines for corporates to facilitate appropriate responses concerning such individuals and of a consultation service with occupational health staff. There are fears that normal medical treatment will be limited or restricted and that disease control will deteriorate, especially for chronic diseases. Therefore, follow‐ups after health checkups must be implemented.

#### 3.4.3 Family members of teleworkers

Involvement with the local community is reduced and social life suffers. While communication with family members may increase and family relationships may improve, individuals may feel frustrated as a result of stress induced by too much proximity. There should be cooperation within families, for example, through shared responsibilities for housework and childcare, and attempts to maintain connections with the external society through interactions with friends via phone calls, emails, or online communication.

### 3.5 Monitoring/evaluation

Of the three occupational health physicians and three occupational health nurses who shared their feedback and inputs on the usefulness of the recommendations. All of these individuals stated that this information helped them to self‐organize. The occupational health physicians opined that they could be used to evaluate which measures are necessary and report instances of utilization, such as checking whether any measures had been omitted and whether they were reflected in educational materials. The occupational health nurses provided the following additional inputs: the measures were “effective and easy to communicate to human resource departments and related departments.” They could be “shared with in‐occupational health staff,” and “the missing perspectives of workers who were not the target of telework” had been addressed.

## 4 DISCUSSION

During the screening process for developing an HIA for evaluating the impacts of the surge of telework in the wake of COVID‐19 in Japan, a number of positive and negative health effects were assumed to apply to teleworkers, non‐teleworkers, and family members of teleworkers. Measures for assessing health effects covering a wide range of subjects were developed for the HIA and were reflected in the corresponding recommendations. We demonstrated the effectiveness of the HIA using a rapid development strategy that entailed formulating a number of useful recommendations within a month of initiating the process and receiving positive feedback on the resulting report from occupational health professionals. Notably, the targeted health effects were partly itemized in the interviews conducted during the appraisal step, and they were mainly based on what was covered in the screening phase, which was an important step in the rapid implementation of an HIA in urgent circumstances.

Studies have shown that telework has many positive health impacts, such as increased work freedom^18^ and more time spent with family members.^19^ One study showed that workers who partially teleworked scored better for obesity and certain lifestyle habits than did non‐teleworkers.^20^ However, telework is also associated with negative effects, such as musculoskeletal disorders, the psychological impacts of isolation and stress, and long working hours.^4^, ^5^ According to one study, health effects relating to musculoskeletal disorders can be reduced through appropriately designed home office environments and targeted ergonomic training.^21^ Isolation and lack of information can lead to overwork and role ambiguity,^22^ and increased social support is an important factor determining successful telework.^23^

Overall, previous studies have indicated that the health effects of telework are essentially positive,^18^, ^19^, ^20^ and that negative health effects,^21^, ^23^ while they do occur, can be mitigated by countermeasures. However, these findings are based on the assumption that telework is an effective work choice and that adequate preparation has preceded its initiation. The negative health aspects are likely to come to the fore when telework is embarked on without any prior preparation. For example, the home office is isolated from the private home environment in the case of telework involving prior preparations, whereas in the current context, entailing inadequate preparation, many workers have been forced to work in their living rooms or at the dining room table. In a context of closures of nursery schools and schools, work‐family conflict rather than the normally positive effect of improved relationships of employees with their family members has been apparent. Thus, when new measures such as telework are introduced, they are often initiated after employees have received training in advance and a suitable work environment has been established. However, in this case, the company, although unprepared, decided to expedite the introduction of telework, so there was a strong possibility that negative health effects would surface. The HIA was conducted after the commencement of the newly proposed project. In principle, an HIA should be conducted before remedial measures are initiated. The disadvantage of doing so, however, is that if the implementation of the proposed measures is expected to take time, they may not be implemented immediately, and the effects of the improvements may not be apparent. One advantage of our approach was that we were able to identify the health impacts already being experienced by stakeholders during the implementation of the HIA. In addition, during a period of rapid change, many individuals tend to deal only with immediate health effects. The HIA provides a broader perspective on the health effects pertaining to an existing situation and will enable the persons in charge to respond in a more relaxed manner.

In this study, we considered three affected populations: teleworkers, non‐teleworkers, and family members of teleworkers. Previous studies have only considered the health effects of teleworkers on telework. Although a company can provide family members of teleworkers just with a limited response, a wider response to the issue as a whole is needed based on the recommendations provided and encompassing non‐teleworkers. During the appraisal process, individuals with special needs were also identified. For example, some occupational physicians noted that workers with mental health issues were particularly affected by mental stress and that individuals with dementia could experience worsening symptoms, which could increase the burden on their families as care providers. Thus, it is also important to consider vulnerable groups who are especially susceptible to negative health impacts.

HIAs are essentially used by particular organizations in their decision‐making process. Accordingly, the recommendations can be directly considered and applied in policy revisions. However, our HIA did not target a specific business; rather it was intended to be applicable to diverse companies. The impact of telework will depend on the characteristics of a business, such as the industry and the worker's occupation, and these factors may affect the applicability of the recommendations. In addition, some of the recommendations entail a certain amount of expenditure for acquiring an appropriate desk, chair, and other components of a suitable working environment at home. Small‐ and medium‐sized businesses experiencing hardship may find it difficult to invest in improving their work environments. In Japan, several ministries and agencies have established subsidies to improve the teleworking environment. Sharing the results of this HIA with government agencies or having government agencies conduct HIAs would provide useful inputs for policy making. Revisions in the rules for personnel would be required for developing a reporting system used at the beginning and end of each workday and for introducing labor management tools. It is also expected that decisions on implementation will vary depending on the degree of continuity of telework after the COVID‐19 pandemic. We propose to use our recommendations in consideration of the priorities and feasibility of implementing the measures. The application of the HIA measures in a crisis, such as the current one, makes it difficult to consider the HIA process comprehensively because the situation and environment are continually and rapidly changing. The last step of the HIA, namely monitoring/evaluation should be ongoing, entailing reviews of the HIA process, as required, and adding or modifying recommendations.

There are limitations relating to the rapid implementation of the HIA in a pandemic crisis. We evaluated the HIA process based on the opinions and impressions of occupational health professionals and not on the adaptation of our recommendations or an assessment of exhaustive health effects. However, in the appraisal process, we succeeded in acquiring real‐time information on the situation from several occupational health physicians at the sites where the countermeasures were being implemented, enabling us to identify common issues faced in many workplaces.

Irrespective of our recommendations, some efforts have already been made to safeguard the health of teleworkers in workplaces where occupational health physicians and nurses are providing guiding inputs and introducing effective practices to improve working conditions. They are sharing their knowledge and experience with other professionals. However, workplaces that lack an occupational health professional may not be able to respond effectively to achieve the expected health effects. In addition, the situation is expected to be prolonged, and the negative effects of telework may become more apparent in the future. Our aim is to promote telework, while implementing additional measures that can contribute to reducing its negative impacts and strengthening its positive ones. Widespread dissemination and utilization of the results of this HIA, conducted at a time of crisis, and continual reviews of the results, combined with the monitoring of the situation within each business unit and the collection of information on the measures that they have been taken, will enhance the positive impacts of telework.

## DISCLOSURE

*Approval of the research protocol:* In the monitoring/evaluation process of this study, we had interviewed three occupational health physicians and three occupational health nurses via email. *Informed Consent*: All were informed and agreed with this study via email. *Registry and the Registration No. of the study/Trial:* N/A. *Animal Studies:* N/A. *Conflict of Interest:* None declared.

## AUTHORS’ CONTRIBUTIONS

TN, DI, MN, YF, and KM (the steering group) conceived of and coordinated the project. TN, AF, and RI conducted the data analysis. The five senior occupational health physicians (KO, SK, MU, IO, and SD) provided inputs on the appraisal of health effects in the health impact assessment. TN, DI, MN, AF, RI, and KM drafted the initial manuscript. TN, MN, YF, and KM revised the manuscript. All of the authors commented on drafts of the manuscript.
